# Prevalence of *Cryptosporidium* spp. oocysts in dogs in Lusaka district of Zambia

**DOI:** 10.14202/vetworld.2018.585-589

**Published:** 2018-05-06

**Authors:** Lamson Mugala, Joyce Siwila, Ngonda Saasa, Girja Shanker Pandey

**Affiliations:** 1 Department of Applied and Health Sciences, Biomedical Section, Evelyn Hone College of Applied Arts and Commerce, Lusaka, Zambia; 2 Department of Clinical Studies, School of Veterinary Medicine, University of Zambia, P. O. Box, 32379, Lusaka, Zambia; 3 Department of Disease Control, School of Veterinary Medicine, University of Zambia, P. O. Box, 32379, Lusaka, Zambia

**Keywords:** *Cryptosporidium* spp, dogs, Lusaka, prevalence, Zambia

## Abstract

**Aim:**

*Cryptosporidium* is one of the causes of diarrheal illness in man and animals worldwide and is zoonotic. The study aimed to determine the prevalence and risk factors associated with fecal shedding of *Cryptosporidium* oocysts in dogs in Lusaka district of Zambia.

**Materials and Methods:**

A cross-sectional study was conducted in Lusaka district of Zambia during 2015-2016. A total of 390 dogs (243 males and 147 females) aged 2 months-13 years were enrolled. Fecal samples were collected and stained using modified Ziehl-Neelsen and Auramine O staining techniques and examined microscopically for oocysts.

**Results:**

Overall, the prevalence of *Cryptosporidium* oocysts infection was 5.9% (23/390; 95% confidence interval: 3.9-8.7). Prevalence among male dogs and female dogs was 5.3% and 6.8%, respectively. Older dogs had a relatively higher infection rate compared to the younger puppies. There was a statistically significant difference in infection between nondescript breed and pure breeds with prevalence being higher in nondescript dog breeds. Water source was also significantly associated with *Cryptosporidium* infection.

**Conclusion:**

*Cryptosporidium* infections are common, especially among the nondescript breed of domestic dogs in Lusaka district of Zambia. Further studies to characterize the common species are warranted.

## Introduction

*Cryptosporidium* is an intracellular zoonotic protozoan parasite that causes cryptosporidiosis, a diarrheal disease of humans and domestic animals. *Cryptosporidium* infections continue to be a significant health problem in both developed and developing countries where it is recognized as an important cause of diarrhea in both immunocompromised and immunocompetent people. Infection may be transmitted from person to person, by direct contact with infected animal or through fecal-oral route by ingestion of oocyst contaminated water and food [1-3]. *Cryptosporidium* infection in dogs occurs when oocysts from the environment are ingested. The prepatent period for *Cryptosporidium* *canis* which specifically infects dogs varies from 2 to 14 days [4]. Infection is more common in dogs of <6 months of age compared to adult dogs. Other than age, overcrowding, especially in breeding areas, is also a risk factor. Most infections are usually asymptomatic [5], but clinical signs such as severe diarrhea, malabsorption, and weight loss have been reported, especially in younger puppies [6].

Even if the zoonotic risk from *Cryptosporidium*-infected dogs is reported to be minimal [7], severely immunocompromised individuals and malnourished children can become ill by infection with *C. canis* [8]. The isolation of *C. canis* from both dogs and children from the same household in Peru also highlighted the possibility of human infections from dogs [9]. The prevalence of *Cryptosporidium* in dogs varies greatly worldwide. Prevalence rates of 0.23-75% have been reported based on different diagnostic methods [1,10-12]. In Zambia, studies on *Cryptosporidium* have been limited to some domestic animals [13,14]; however, only one of these studies included dogs, no *Cryptosporidium* oocysts were detected in the only 20 adult dogs that were examined [15].

This study, therefore, was aimed to determine the occurrence and risk factors associated with fecal shedding of *Cryptosporidium* oocysts in dogs, covering a larger sample size in selected areas of Lusaka district of Zambia.

## Materials and Methods

### Ethical approval

This study complied with guidelines laid down by the Institutional Ethics Committee and in accordance with country’s law.

### Study area

This study was conducted in three purposively selected veterinary clinics and two high-density residential areas, namely, Kabanana and Kalingalinga, all situated in Lusaka district of Zambia. The district has a total human population of 1,747,152 with about 358,871 households.

### Study design and sampling

The study was conducted between October 2015 and May 2016. All the dogs presented to selected veterinary clinics were sampled (except those presenting with suspected parvovirus enteritis), and samples were also collected from dogs presented for vaccination from two residential areas during a vaccination campaign against rabies. The sample size was estimated using the simple random formula [16]. Due to lack of a previous estimate of *Cryptosporidium* in dogs in Zambia and to get the maximum sample size, the prevalence was estimated at 50% with a margin of error of 0.05 and a confidence level of 95%. A total of 390 dogs were included in the study.

A structured questionnaire was used to capture demographic information of the dogs and management factors such as feeding habits, whether the dog was enclosed or not, how many dogs were kept in the household and water sources among others. A single fecal sample was collected *per rectum* from each dog using individual disposable latex glove, which was tied off and marked with the dog’s identity number and placed in a cool box packed with ice. The samples were transported to the University of Zambia, School of Veterinary Medicine Laboratory where they were processed the same day.

### Laboratory sample analysis

Duplicate fecal smears were prepared on a glass slide from each sample. Modified Ziehl-Neelsen (MZN) [17] and Auramine O staining methods [18] were used to stain and demonstrate the presence of *Cryptosporidium* oocysts in the fecal samples. Slides were examined microscopically. A sample was considered positive if at least one *Cryptosporidium* oocyst was visualized and identified as a pinkish-red oocyst against a blue background.

### Statistical analysis

Data were entered using a Microsoft Excel^®^ spreadsheet, and all analyses were performed using Statistical Package for Social Sciences version 16.0. Proportions of positives, with 95% confidence intervals, were estimated. The relationships between parasite presence and age, sex of dog, and other hypothesized risk factors were determined using Chi-square or Fisher’s exact test where appropriate. A significance level of 5% was used for all analyses.

## Results

### Prevalence of *Cryptosporidium*

**Figure-1:**
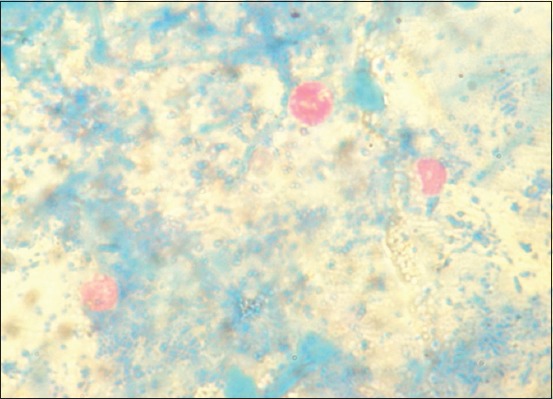
*Cryptosporidium* oocysts stained with modified Ziehl-Neelsen staining (100X).

Of the 390 fecal samples (22 diarrheic and 368 non-diarrheic), 5.9% (95% confidence interval [CI]: 3.9-8.7) tested positive for *Cryptosporidium* oocysts (Auramine O), all dogs being asymptomatic. Microscopic examination with MZN staining revealed pink stained round *Cryptosporidium* oocysts against a blue background (Figure-1); the prevalence of *Cryptosporidium* was 5.4% (21/390; 95% CI: 3.5-8.1).

The prevalence of *Cryptosporidium* (Auramine O) according to collection sites was 4.3% (9/206; 95% CI: 2.3-8.2) for clinic samples while that for residential (Kalingalinga and Kabanana) areas were 8.8% (6/68; 95% CI: 3.9-18.6) and 6.9% (8/116; 95% CI: 3.4-13.3), respectively. Although the prevalence of *Cryptosporidium* infection was relatively higher in Kalingalinga residential area (8.8%), there was no statistically significant difference among the three sample sources (p=0.331). When the prevalence of *Cryptosporidium* was compared between clinic and residential samples, there was also no statistically significant difference in the infection rates (p=0.158).

The prevalence of *Cryptosporidium* on MZN based on the sampling sites was 3.4% (7/206; CI: 1.6-6.9) for clinic samples compared to 7.6% (14/184; 95% CI: 4.5-12.5) for residential areas; with Kalingalinga having a prevalence of 8.8% (8/116; 95% CI: 3.9-18.6) while that for Kabanana was 6.9% (6/68; 95% CI: 3.4-13.3). Similar to Auramine O, results were not significantly different on MZN (p=158).

### Sex- and age-related prevalence

Of the 147 female dogs sampled, 10 (6.8%) were found to have *Cryptosporidium* spp. oocysts and 5.3% (13/243) male dogs were positive for *Cryptosporidium* oocysts. The difference was, however, not statistically significant on both MZN (p=0.658) and Auramine O (p=0.360) (Table-1).

**Table-1:** Sex, age, breed, and water source-related distribution of Cryptosporidium infection in dogs in Lusaka (n=390).

Variable	Number tested*	Positive (%)	p-value
Sex			
Males	243	13 (5.3)	0.658
Females	147	10 (6.8)	
Age (months)			
2-6	78	3 (3.8)	0.611
7-12	67	3 (4.5)	
>12	245	17 (6.9)	
Total	390	23 (5.9)	
Breed			
Nondescript	280	20 (7.1)	0.012
Pure breed	110	1 (0.9)	
Total	390	21 (5.4)	
Water source			
Municipal	284	21 (7.4)	0.041
Borehole	106	2 (1.9)	
Total	390	23 (5.9)	

* Auramine O

The age range of the dogs was from 2 months to 13 years with majority being above 1 year. Most dogs were below 6 years of age. Of the puppies from 2 to 6 months of age, only 3.8% (3/78) were found positive for *Cryptosporidium*. The number of positive dogs in the juvenile group (7-12 months) was similar (4.5%; 3/67) to that in puppies, leaving the older dogs with a higher prevalence (6.9%; 17/246) (Table-1). The difference was, however, not statistically significant on both Auramine O (p=0.611) and MZN (p=0.808).

### Prevalence of *Cryptosporidium* spp. according to breed

Most of the dogs included in the study were of nondescript breed (280 vs. 110). The prevalence of *Cryptosporidium* infection was higher among nondescript breed of dogs than in pure breeds, though not statistically significant (p=0.149). However, under the MZN stain, there was a statistically significant difference in *Cryptosporidium* infection between nondescript breed and pure breed dogs (p=0.012). The prevalence was significantly higher in nondescript dogs (7.1%; 20/280) compared to purebreds (0.9%; 1/110).

### Dog keeping practices and prevalence of *Cryptosporidium* spp.

During sample collection, dog owners were asked what type of food they fed the dogs, whether the dogs were enclosed or not, the water source for the animals and whether their dog(s) had experienced diarrhea in the past 3 weeks before the study. Most of the dogs were fed kitchen leftover foods (348/390) while the rest were either fed commercial dog food or a combination of kitchen leftovers and commercial dog food. A few (2%; 8/390) dogs were fed meat saw dust. There was, however, no relationship between type of food fed and *Cryptosporidium* infection in the dogs (p=0.999). There was also no statistically significant difference in infection rate whether the dogs were enclosed or not (p=0.254) and whether they had experienced a diarrhea episode or not (p=0.631). On the other hand, water source was found to be associated with *Cryptosporidium* infection in the dogs (p=0.041) (Table-1).

## Discussion

The prevalence of *Cryptosporidium* spp. in dogs in Lusaka district was determined in this study, with an overall prevalence of 5.9%. Other studies from elsewhere have confirmed the presence of *Cryptosporidium* in dogs with variations in prevalence. Climatic and seasonality differences and the laboratory techniques used could explain the variations. A study in humans in Zambia highlighted the influence of season on the prevalence of *Cryptosporidium* spp. [19]; an indication that rainy season undoubtedly increases environmental transport of contaminated feces and/or materials. Effect of climate and seasonality on *Cryptosporidium* infection in dogs would provide great insights into the transmission dynamics of the parasite; this will be explored in future studies. The prevalence in this study (5.9%) was lower than the 16% reported in Ontario Canada [20], 75% reported in Costa Rica [12], and 44% reported in South Africa [1]. However, low prevalence levels have also been reported previously such as 3.8% in Henan Province, China [21].

*Cryptosporidium* prevalence varied from one residential area to another; it was relatively higher in Kalingalinga (8.8%) compared to Kabanana (6.9%). Both are densely populated with most dogs that are kept being semi-stray. *Cryptosporidium* prevalence from the dogs sampled from the clinics was much lower. This cohort of dogs probably receives better care and clean/safe food and treated water compared with those from the field which often scavenge for food and water.

The sex of the dog had no influence on *Cryptosporidium* infection, as reported by Jian *et al*. [21]; although the prevalence was relatively higher in female dogs than in male dogs in the current study. A similar comparative study in Nigeria indicated that female dogs were more likely to contract intestinal protozoa than male dogs [11]. Zelalem *et al*. [22] reported a higher *Cryptosporidium* prevalence in male (79.2%) than female (76.8%) dogs. The higher prevalence in females reported in the current and others studies [22] could be due to reduced immunity at certain periods in females’ physiologic cycle [11]. However, given the ubiquitous nature of *Cryptosporidium*, both sexes have equal chances of getting infected if they are exposed to the infected material.

According to age, older dogs had a relatively higher prevalence of *Cryptosporidium* compared to young ones but with no significant difference. The findings are in contrast with those of Tangtrongsup *et al*. [4] and Ramirez *et al*. [5] who reported that young dogs are more likely to be parasitized than adults. Young puppies are said to be more susceptible to various infections including *Cryptosporidium* due to undeveloped immunity unlike older dogs [23], the present study, to the contrary, found a higher infection rate in older animals. Gbemisola *et al*. [11] attributed a high *Cryptosporidium* infection rate in older dogs to the use of older dogs for security purposes, thereby increasing their tendency to move around more often and possibly getting infected, which could also explain the current findings. However, the number of adult dogs tested in this study was much higher than that for younger dogs. Moreover, puppies are less likely to scavenge and therefore would probably be less exposed to different sources of contamination.

Breed was found to be significantly associated with the prevalence of *Cryptosporidium* infection with more cases reported among nondescript dogs than in pure breeds. Our findings are consistent with those of Adejimi and Osayomi [24]. Contrary findings were reported by Awadallah and Salem [25] from a study in Egypt which did not find any relationship between dog breed and *Cryptosporidium* infection. Majority of the dogs in the current study were semi-stray and were, therefore, more likely to be exposed to contaminated areas/food and water which could have been contaminated with *Cryptosporidium* oocysts.

*Cryptosporidium* infection was only detected in dogs without diarrhea. This is in agreement with previous reports indicating that most infections in dogs are asymptomatic [5], but infected dogs continue shedding oocysts. Diarrhea in symptomatic dogs was probably due to other pathogens. It has not been shown previously that diarrhea in dogs (especially puppies) can solely be caused by *Cryptosporidium* infection as dogs that were previously reported to have diarrhea and were positive for *Cryptosporidium* infections had other concurrent infections (parvovirus enteritis or canine distemper) [6].

It has been reported previously that outdoor cats and dogs are approximately 5 times more likely to be infected with *Cryptosporidium* species than indoor ones [10]. Higher prevalence of *Cryptosporidium* in non-enclosed dogs in the current study could be due to scavenging which can lead to consumption of contaminated food or drinking contaminated water. Water source was also found to be associated with infection in dogs from households using municipal water source. Previous studies in humans [26] and water supplies [27] in Lusaka, Zambia, reported water contamination to have a major influence on infection, the water source being similar to that investigated in the current study. *Cryptosporidium* is reported to be resistant to chlorination [10,28] which is commonly used to treat municipal water locally. However, infected dogs in this study could have acquired the infection from other sources.

The number of dogs and other types of animals kept per household did not influence *Cryptosporidium* infection contrary to other researchers who indicated that overcrowding of dogs and constant contact with other animals such as cats could contribute to high prevalence of *Cryptosporidium* infection in dogs [1]. Further, diet was not a risk factor unlike Abere *et al*. [29], who found an association between the prevalence of *Cryptosporidium* and feeding management. Most respondents did not know the recommended number of dogs to be kept per household as stipulated under chapter 247 of 1994 of the laws of Zambia. Compliance with this law would assist in controlling dog populations and ensuring that owners only keep the number of dogs that they can manage and care for. Furthermore, it would assist in reducing the number of stray or semi-stray dogs, hence, preventing the potential spread of infectious and zoonotic pathogens.

## Conclusion

The present study has shown that *Cryptosporidium* spp. infections are present among dogs in Zambia, more so in asymptomatic form, and that nondescript dogs are more commonly infected compared to pure breed dogs. Although financial constraints did not permit molecular work in this study, future studies will consider inclusion of molecular techniques, wider area coverage, as well as climatic and seasonality patterns of infection.

## Authors’ Contributions

LM and JS contributed to the conception/design of the study. LM and JS conducted the data collection and analysis. NS and GSP participated in the data analysis. JS prepared the first draft and GSP and NS participated in the correction of the manuscript. All the authors have read and approved the final manuscript.
